# Romosozumab Enhances Vertebral Bone Structure in Women With Low Bone Density

**DOI:** 10.1002/jbmr.4465

**Published:** 2021-12-16

**Authors:** Kenneth ES Poole, Graham M Treece, Rose A Pearson, Andrew H Gee, Michael A Bolognese, Jacques P Brown, Stefan Goemaere, Andreas Grauer, David A Hanley, Carlos Mautalen, Chris Recknor, Yu‐Ching Yang, Maria Rojeski, Cesar Libanati, Tristan Whitmarsh

**Affiliations:** ^1^ Department of Medicine University of Cambridge and Addenbrooke's Hospital Cambridge UK; ^2^ Department of Engineering University of Cambridge Cambridge UK; ^3^ Bethesda Health Research Center Bethesda MD USA; ^4^ CHU de Québec Research Centre and Laval University Quebec City Canada; ^5^ Ghent University Hospital Ghent Belgium; ^6^ Amgen Inc. Thousand Oaks CA USA; ^7^ University of Calgary Calgary Canada; ^8^ Centro de Osteopatias Medicas Buenos Aires Argentina; ^9^ United Osteoporosis Centers Gainesville GA USA; ^10^ UCB Pharma Brussels Belgium; ^11^ Institute of Astronomy, University of Cambridge Cambridge UK

**Keywords:** BONE QCT/microCT, ANALYSIS/QUANTITATION OF BONE, OSTEOPOROSIS, DISEASES AND DISORDERS OF/RELATED TO BONE, ANABOLICS, THERAPEUTICS, ANTIRESORPTIVES

## Abstract

Romosozumab monoclonal antibody treatment works by binding sclerostin and causing rapid stimulation of bone formation while decreasing bone resorption. The location and local magnitude of vertebral bone accrual by romosozumab and how it compares to teriparatide remains to be investigated. Here we analyzed the data from a study collecting lumbar computed tomography (CT) spine scans at enrollment and 12 months post‐treatment with romosozumab (210 mg sc monthly, *n* = 17), open‐label daily teriparatide (20 μg sc, *n* = 19), or placebo (sc monthly, *n* = 20). For each of the 56 women, cortical thickness (Ct.Th), endocortical thickness (Ec.Th), cortical bone mineral density (Ct.bone mineral density (BMD)), cancellous BMD (Cn.BMD), and cortical mass surface density (CMSD) were measured across the first lumbar vertebral surface. In addition, color maps of the changes in the lumbar vertebrae structure were statistically analyzed and then visualized on the bone surface. At 12 months, romosozumab improved all parameters significantly over placebo and resulted in a mean vertebral Ct.Th increase of 10.3% versus 4.3% for teriparatide, an Ec.Th increase of 137.6% versus 47.5% for teriparatide, a Ct.BMD increase of 2.1% versus a −0.1% decrease for teriparatide, and a CMSD increase of 12.4% versus 3.8% for teriparatide. For all these measurements, the differences between romosozumab and teriparatide were statistically significant (*p* < 0.05). There was no significant difference between the romosozumab‐associated Cn.BMD gains of 22.2% versus 18.1% for teriparatide, but both were significantly greater compared with the change in the placebo group (−4.6%, *p* < 0.05). Cortical maps showed the topographical locations of the increase in bone in fracture‐prone areas of the vertebral shell, walls, and endplates. This study confirms widespread vertebral bone accrual with romosozumab or teriparatide treatment and provides new insights into how the rapid prevention of vertebral fractures is achieved in women with osteoporosis using these anabolic agents. © 2021 The Authors. *Journal of Bone and Mineral Research* published by Wiley Periodicals LLC on behalf of American Society for Bone and Mineral Research (ASBMR).

## Introduction

Several licensed treatments for osteoporosis are considered “anabolic” because they stimulate bone accrual in osteoporotic vertebrae. Teriparatide is a peptide comprising the amino‐terminal 34 amino acids of human parathyroid hormone. It enhances bone remodeling with a positive ulterior bone balance.^(^ ^1^ ^)^ The monoclonal antibody romosozumab,^(^ ^2^ ^)^ on the other hand, stimulates osteoblastic activity while reducing osteoclastic activity by binding and inhibiting the action of sclerostin, an osteocyte‐derived inhibitor of bone formation.^(^ ^3^ ^)^ The early dual effect of this is the rapid formation of new bone by modeling on predominantly the endocortical and cancellous envelopes, with a decrease in bone resorption.^(^ ^4^, ^5^, ^6^ ^)^ Here we examined the effects of 12 months of romosozumab or open‐label teriparatide on the first lumbar vertebra (L_1_) of postmenopausal women with low bone mass in a phase 2, international, randomized, placebo‐controlled trial (RCT).^(^ ^2^ ^)^

Various imaging techniques have previously been used to demonstrate gains in bone density, thickness, and simulated vertebral strength in response to romosozumab and teriparatide.^(^ ^2^, ^7^, ^8^, ^9^, ^10^, ^11^ ^)^ Understanding where bone is augmented by osteoporosis therapy is important because the mechanism of osteoporotic vertebral fracture, such as anterior wedge deformity or endplate concavity, depends not only on the manner in which forces are applied^(^ ^12^, ^13^, ^14^ ^)^ but also on local deficiencies in bone structure.^(^ ^15^, ^16^ ^)^ Determining the localization of new bone deposition is therefore of great clinical relevance. A recent clinical biopsy study involving romosozumab identified effects in two key compartments: a 328% greater bone formation rate in cancellous bone and a 233% higher rate on the endocortical surface at the ilium after only 2 months' therapy.^(^ ^5^ ^)^ The cortical thickness was correspondingly higher than placebo at 12 months. In the current study, we analyze computed tomography (CT) data of the L_1_ vertebrae using cortical bone mapping,^(^ ^17^, ^18^, ^19^, ^20^, ^21^ ^)^ a validated 3‐dimensional technique to identify the amount and distribution of added bone mass. Statistical parametric mapping (SPM) is subsequently used to analyze the changes in bone mass across the vertebral bone surface, while accounting for differences between individual vertebral shapes and correcting for multiple comparisons.^(^ ^22^ ^)^

In this study, the first lumbar vertebrae were examined using standard clinical CT in untreated postmenopausal women with low bone mineral density at baseline and 1 year later during a phase 2 clinical study. Participants were administered romosozumab under placebo‐controlled double‐blinded conditions, with a third group randomized to open‐label teriparatide. Cortical parameter changes were measured for the entire vertebral bone surface. Color mapping was subsequently used to display the changes across the bone surface in 3D. This work augments prior studies that described the gains in strength, density, and mass with these therapies.^(^ ^8^, ^9^, ^11^ ^)^

## Materials and Methods

### Cortical parameter mapping

In the past years, several methods have been developed to estimate cortical and trabecular parameters from CT images, such as thresholding based^(^ ^23^ ^)^ or using the full‐width half‐maximum method.^(^ ^24^ ^)^ These methods tend to be unreliable when the cortex is thin relative to the imaging resolution. To overcome some of these limitations, a deconvolution‐based method was previously proposed, which has consistently been shown to outperform traditional methods.^(^ ^17^, ^18^, ^25^ ^)^ By making assumption about the shape of the cortex, this deconvolution‐based method is able to estimate the cortical parameters below the spatial resolution of the image.

**Fig. 1:**
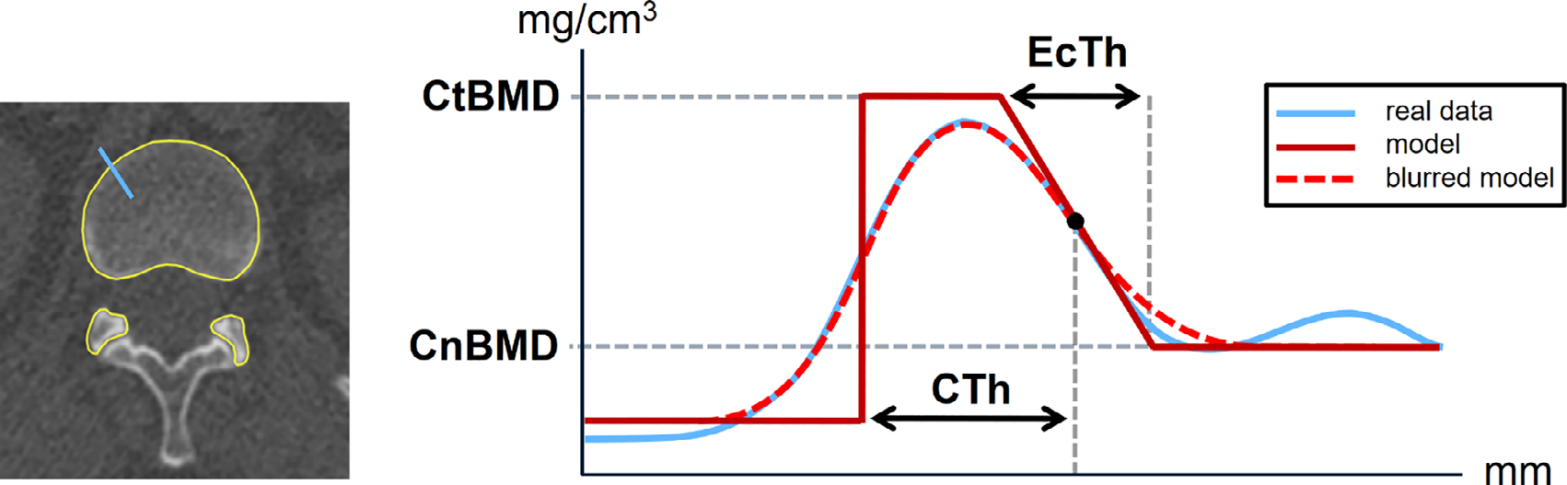
A schematic visualization of the cortical measurement process. Left: A CT slice with the surface mesh shown in yellow and the sampling line in blue. Right: The registration of the blurred cortical model, which is described by the various cortical parameters, onto the real data.

However, measuring true cortical thickness even in high‐resolution micro‐CT images can be challenging, since areas of high porosity, double‐shell cortices, and indistinct cortices are all recognized histologically but blurred when imaged using CT imaging, particularly in the clinical setting.^(^ ^26^, ^27^, ^28^, ^29^ ^)^ Moreover, the transition point from cortical bone to endocortical trabecular bone is often not well defined. A recent advance to address this issue is the ability to assign a separate thickness to the endocortical bone by modeling its shape as a slope of linearly decreasing density,^(^ ^30^ ^)^ as shown in schematic form in Fig. 1. For this, a modification was made to the newest implementation of Stradwin (now known as Stradview), which allows for the evaluation of treatment‐associated changes within a defined endocortical compartment.

The method requires the segmentation in the form of a triangular surface mesh of the vertebrae in each CT scan, which are generated using the Stradwin interface. At each vertex, Stradwin then samples the CT values perpendicular to the bone surface. This results in a profile of the CT values that has an inherent smoothness. By fitting a blurred model of the cortex to the data samples, Stradwin is able to accurately estimate the cortical parameter, as illustrated in Fig. 1. Here the blue line represents the real CT data samples. The red line depicts the model of the cortex described by the cortical parameters and the dashed red line is the blurred model. Stradwin finds the parameters of the model such that the blurred model best fits the real data. This provides us with a measurement of the cortical bone mineral density (Ct.bone mineral density (BMD)) and the cancellous BMD (Cn.BMD) directly adjacent to the endocortex. The slope relates to the endocortical transitional region between the cortex and the cancellous bone. The size of the slope thus defines the endocortical thickness (Ec.Th). In contrast to the method previously published,^(^ ^30^ ^)^ the region that defines the cortical thickness (Ct.Th) reaches from the periosteal surface to the midpoint of the endocortical region. Thus, the cortex and endocortex will partly overlap (as they do histologically) in the model, as shown in Fig. 1.

Defining the cortex in this way has shown to provide robust measurements of the cortical thickness, as confirmed by the evaluation described in Appendix S1. Unlike cortical measures, endocortical and cancellous measures cannot be calculated within the spinous processes and pedicles of the vertebrae because of the close proximity of the opposing cortices. Thus, for the Cn.BMD and Ec.Th, the values and maps are only reported for the vertebral body. Finally, from the cortical thickness and Ct.BMD, we compute the cortical mass surface density (CMSD) as CMSD = 0.1 × Ct.Th × Ct.BMD. This is the mass per unit surface area and is defined in milligrams per square centimeter (mg/cm^2^). It is best interpreted as the mass of the cortical and endocortical bone projected onto the bone surface. Stradwin measures these parameters at each point on the bone surface, resulting in a color‐coded map of the cortical parameters values. In addition, we apply a smoothing over the cortical parameters across the bone surface.^(^ ^31^ ^)^ This serves as a noise removal, as well as provides a value at the vertex in case the model failed to fit to the data.

### Data

To examine the effects of romosozumab and teriparatide treatment, data were analyzed from a QCT substudy of a randomized, multi‐national, placebo‐controlled parallel‐group phase 2 study (ClinicalTrials.gov: NCT00896532) of postmenopausal women aged 55 to 85 years. Inclusion criteria were mainly based on dual‐energy X‐ray absorptiometry (DXA) *T*‐scores in the range of ≤−2.0 and ≥−3.5. The full study criteria and details of the scanning protocol are available from the prior studies published on these data,^(^ ^9^, ^11^ ^)^ which followed up from the preceding phase 1b trial as described in Graeff and colleagues.^(^ ^8^ ^)^ The study protocol was approved by an independent ethics committee or institutional review board at each study site before the study started. All subjects provided written, informed consent to participate in the trial.

QCT scans were acquired at 15 centers with whole‐body spinal CT scanners with at least four detector rows. The same QCT scanner was used for baseline and follow‐up (12‐month) scans of each subject. Participants lay on a Mindways calibration phantom (Mindways Software Inc., Austin, TX, USA) to facilitate accurate conversion of Hounsfield units to bone mineral density values. Scans were performed at 120 kV and were reconstructed with a 1.0 mm (or 1.25 mm) slice thickness. Subjects were only included in the analysis reported here when L_1_ was evaluable in both baseline and follow‐up CT. Patients received blinded romosozumab sc injections 210 mg monthly (*n* = 17), placebo sc injections monthly (*n* = 20), or open‐label sc teriparatide 20 mg daily (*n* = 19). All received 1000 mg calcium and 800 IU vitamin D daily by mouth. Table 1 shows the full set of baseline characteristics. There are no statistically significant differences between treatment groups for any of the measurements as assessed by one‐way ANOVA tests.

**Table 1:** Baseline Demographics and Characteristics (Mean ± Standard Deviation)

	Placebo (*n* = 20)	Teriparatide (*n* = 19)	Romosozumab (*n* = 17)
Age (years)	66.8 ± 6.1	65.2 ± 6.1	64.2 ± 5.0
Weight (kg)	62.8 ± 7.8	66.5 ± 13.9	66.0 ± 12.3
Height (cm)	155.1 ± 6.7	156.5 ± 8.6	155.4 ± 6.8
*T*‐score lumbar spine	−2.26 ± 0.43	−2.30 ± 0.51	−2.59 ± 0.40
Mean Ct.Th (mm)	0.93 ± 0.08	0.91 ± 0.07	0.89 ± 0.07
Mean Ct.BMD (mg/cm^3^)	787.7 ± 53.7	799.3 ± 55.0	793.6 ± 51.2
Mean Cn.BMD (mg/cm^3^)^a^	112.2 ± 17.7	118.4 ± 20.4	113.3 ± 24.7
Mean Ec.Th (mm)^a^	0.091 ± 0.051	0.094 ± 0.042	0.087 ± 0.050
Mean CMSD (mg/cm^2^)	74.1 ± 9.5	73.4 ± 8.0	71.1 ± 4.1

Ct.Th = cortical thickness; Ct.BMD = cortical bone mineral density; Cn.BMD = cancellous bone mineral density; Ec.Th = endocortical thickness; CMSD = cortical mass surface density.

There are no statistical between‐group differences for any of the measurements as assessed by one‐way ANOVA tests.

^a^ Cn.BMD and Ec.Th values are of the vertebral body only.

### Statistical analysis

**Fig. 2:**
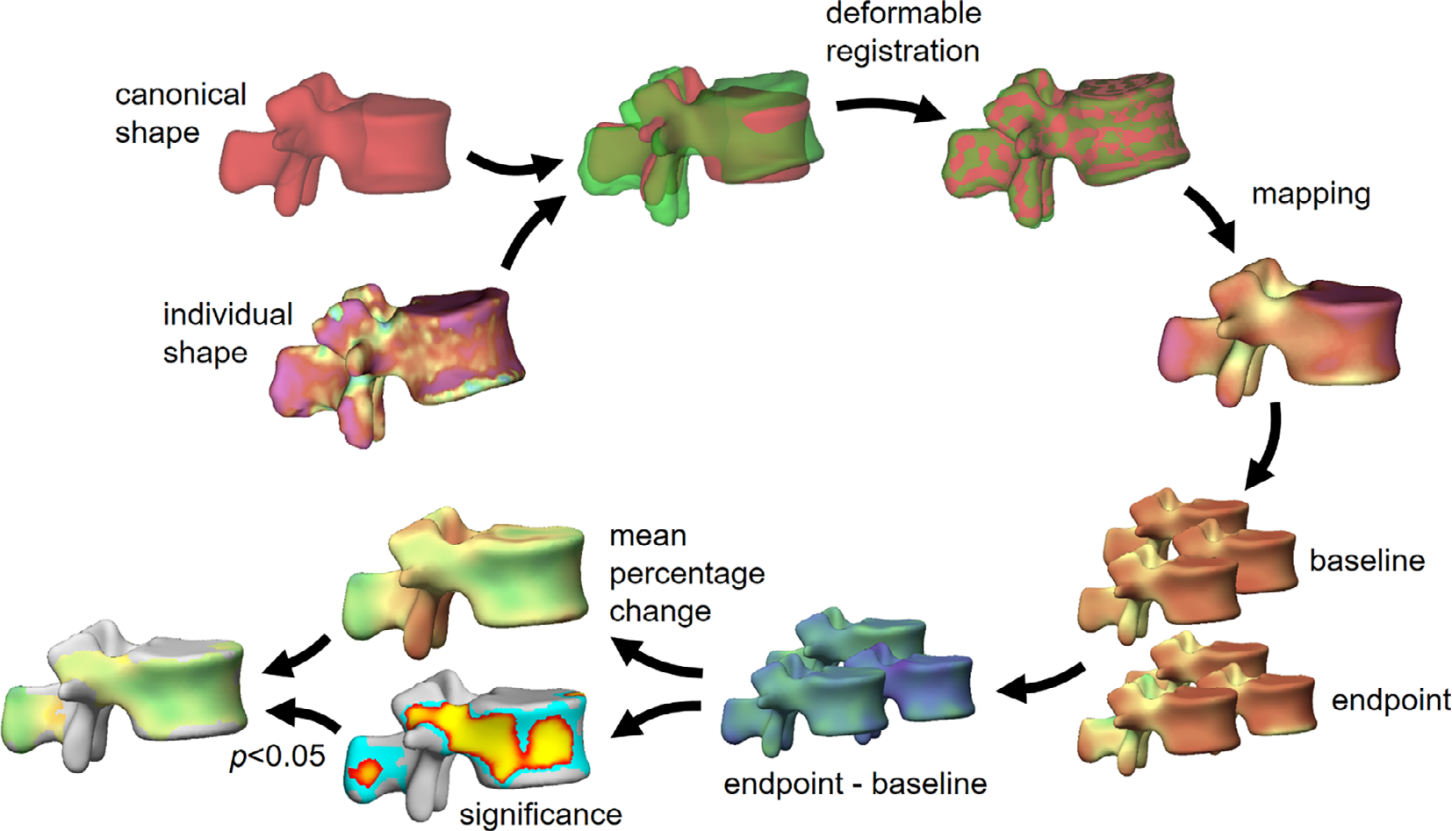
The cortical parameter mapping pipeline.

For the statistical analysis of the cortical measurements, we use a technique called cortical bone mapping,^(^ ^31^ ^)^ which is represented visually in Fig. 2. Briefly, a cortical map was produced for each of the parameters for every CT scan. Next, each cortical map was registered onto a canonical L_1_ vertebral shape using a deformable registration. After this registration, all the cortical parameter measurements were transferred to the vertices in the canonical shape to give one‐to‐one correspondence between the cortical parameters of all the vertebrae. For this longitudinal study, cortical maps were created from L_1_ vertebra at baseline and at the 12‐month endpoint. The global cortical percentage changes with respect to baseline were then calculated for each treatment group (Table 2). Statistical parametric mapping subsequently produced a color‐coded map of the mean percentage changes, as well as a map showing the localized significances of the changes. For the results, the two maps were combined into one where we make the regions gray where the changes were not statistically significant.

**Table 2:** Mean (± Standard Deviation) Percentage Changes From Baseline at 12 Months of the Cortical Parameters for Each Treatment Group

	Placebo (*n* = 20)	Teriparatide (*n* = 19)	Romosozumab (*n* = 17)
Ct.Th	0.6 ± 3.4	4.3 ± 3.4* ^,^ **	10.3 ± 4.9* ^,^ ** ^,^ ***
Ct.BMD	−0.3 ± 2.6	−0.1 ± 2.8	2.1 ± 3.3* ^,^ ** ^,^ ***
Cn.BMD^a^	−4.6 ± 6.1*	18.1 ± 14.4* ^,^ **	22.2 ± 6.6* ^,^ **
Ec.Th^a^	8.2 ± 29.7	47.5 ± 34.5* ^,^ **	137.6 ± 80.5* ^,^ ** ^,^ ***
CMSD	0.2 ± 2.0	3.8 ± 2.7* ^,^ **	12.4 ± 4.7* ^,^ ** ^,^ ***

Ct.Th = cortical thickness; Ct.BMD = cortical bone mineral density; Cn.BMD = cancellous bone mineral density; Ec.Th = endocortical thickness; CMSD = cortical mass surface density.

^a^ Cn.BMD and Ec.Th values are of the vertebral body only.

* *p* ≤ 0.05 versus baseline.

** *p* ≤ 0.05 versus placebo.

*** *p* ≤ 0.05 versus teriparatide, using two‐tailed *t* tests.

## Results

The primary outcomes of the study were the percentage change with respect to baseline in Ct.Th, Ct.BMD, Ec.Th, Cn.BMD, and CMSD in each group. Table 2 shows that by 12 months, romosozumab improved all parameters significantly over placebo and resulted in a mean vertebral Ct.Th increase of 10.3% ± 4.9% versus 4.3% ± 3.4% for teriparatide, a Ct.BMD increase of 2.1% ± 3.3% versus −0.1% ± 2.8%, and an Ec.Th increase of 137.6% ± 80.5% versus 47.5% ± 34.5% for teriparatide, with all differences statistically significant. The Cn.BMD increase of 22.2% ± 6.6% with romosozumab versus 18.1% ± 14.4% with teriparatide was not statistically significantly different. For the placebo group, there was no statistically significant change, except for Cn.BMD, which decreased by −4.6% over 12 months.

**Fig. 3:**
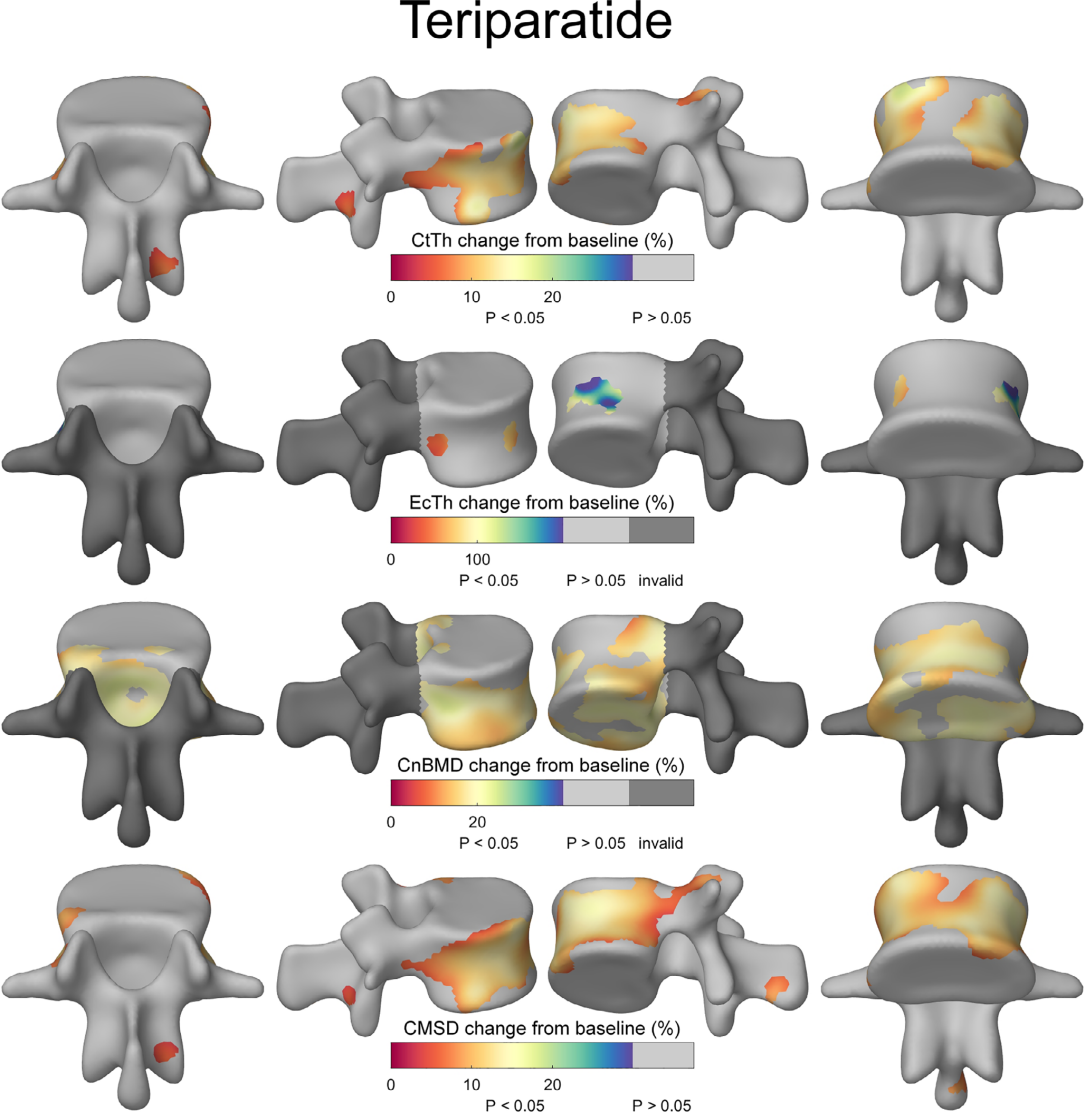
Mean percentage changes from baseline after 12‐month treatment of teriparatide measured by cortical bone mapping. Ct.BMD is not displayed because of the lack of regions with significant changes. Light gray regions had no statistically significant changes with time. Dark gray regions of the spinous processes and pedicles were not examined for endocortical and cancellous parameters.

**Fig. 4:**
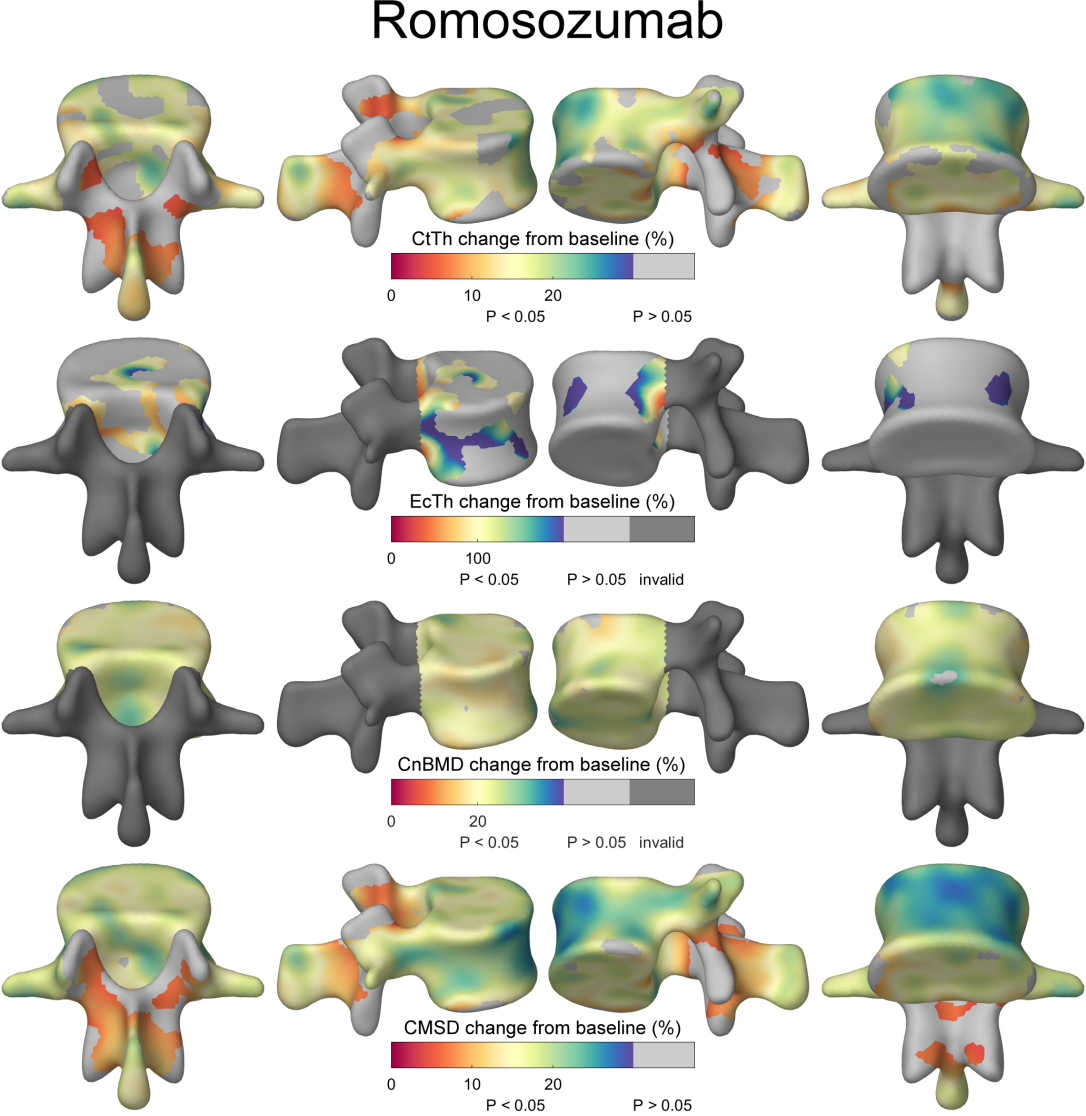
Mean percentage changes from baseline after 12‐month treatment of romosozumab measured by cortical bone mapping. Ct.BMD is not displayed because of the lack of regions with significant changes. Light gray regions had no statistically significant changes with time. Dark gray regions of the spinous processes and pedicles were not examined for endocortical and cancellous parameters.

The cortical maps in Figs. 3 and 4 represent the % difference at 12 months with the light gray regions indicating no significant change with time. They show the topographical locations of the increase in Ct.Th, Ec.Th, Cn.BMD, and CMSD in response to teriparatide (Fig. 3) and romosozumab (Fig. 4) treatment. The corresponding figures with the absolute changes are shown in Supplemental Figs. S3 and S4. The results indicate an increase of Ct.Th, Ct.BMD, and CMSD predominantly at the vertebral shell in the teriparatide‐treated group, while romosozumab resulted in an overall increase, including in the fracture‐prone areas of the vertebral shell and endplates.

## Discussion

Various imaging techniques have previously been used to demonstrate gains in bone density and thickness using romosozumab and teriparatide trial data.^(^ ^2^, ^7^, ^8^, ^9^, ^10^, ^11^ ^)^ Our results are complementary to these prior studies, with the added contributions being global and accurate measures of the cortical bone changes, and the spatial mapping of areas of statistically significant bone accrual. Supplemental Fig. S2 indicates that cortical bone mapping using clinical CT data is feasible across the range of typical vertebral thicknesses in older individuals. It is noteworthy that the measurements in our sample of 20 vertebral bodies of the baseline scans in the placebo group have an average thickness of 0.67 ± 0.13 mm, which is in agreement with the histological measurements made by Fazzalari and colleagues^(^ ^32^ ^)^ (mean 0.47 ± 0.32 mm) when a more representative sampling method for histological assessment of thin and thicker vertebral areas was done.

We show a small yet statistically significant increase in Ct.BMD of 2.1% in the romosozumab group and a nonsignificant decrease in the teriparatide group of −0.1%. QCT data from the same clinical trial has been published before using established methods for measuring bone changes in the cortical and trabecular compartments.^(^ ^9^ ^)^ However, such compartmental‐based analysis tends to overestimate the cortical region. Consequently, the cortical changes shown in Genant and colleagues^(^ ^9^ ^)^ will also carry some of the larger cancellous bone changes. Our proposed deconvolution method, on the other hand, allows for a robust separation of the cortical from the endocortical changes. Considering these methodological differences, we cannot directly compare our Ct.BMD changes with those provided by Genant and colleagues.^(^ ^9^ ^)^

Represented graphically as the area under the curve in Fig. 1, the CMSD consolidates cortical thickness, density, and endocortex into a measure of cortical mass at each vertex. We show average CMSD increases of 3.8% and 12.4% for teriparatide and romosozumab groups, respectively. With the CMSD value being independent of the way in which the cortical bone is separated from the endocortical bone, CMSD is our most reliable quantitative measure of the cortical bone accrual. When comparing two treatment types with different effects on bone volume and density, care has to be taken when interpreting and comparing the measured changes in individual cortical parameters. Romosozumab has a dual action, increasing modeling‐based bone formation and reducing resorption. The initial, rapid modeling‐based effects are to deposit additional surface osteoid, without any increase in cortical porosity.^(^ ^5^ ^)^ Conversely teriparatide has been shown to initially decrease BMD by enhancing remodeling‐based resorption, resulting in a transiently more porous cortex. At the same time, newly deposited osteoid from the stimulation of remodeling‐based (and to a lesser extent modeling‐based) formation occurs, increasing the eventual cortical volume/thickness by endocortical apposition. With a compartmental analysis, it will be difficult to separate the two in clinical CT scans due to the limited resolution. This will especially be true if the cortices are very thin or when the changes are rather small, such as those expected after a treatment period of only 12 months. Using the previous deconvolution method using a step‐based model, we hypothesized that the above described effect is measured as either a large increase in thickness with decrease in density or a smaller increase in thickness with an increase in endocortical trabecular density, with the final measure being an average of the both.^(^ ^28^ ^)^ With our novel approach, we attempt to reduce the ambiguity by also modeling the shape of the endocortical region. We consider that this more accurately describes the genuine underlying morphological changes happening in the cortex due to the various treatment options.

Other studies on the effects of romosozumab also show similar increases in cortical thickness and trabecular BMD. In Graeff and colleagues,^(8^ ^)^ a 3‐month administration of romosozumab resulted in an increased trabecular BMD of 10.3% ± 1.4% and 9.5% ± 1.5% as measured from QCT and high‐resolution QCT (HR‐QCT), respectively. Trabecular bone volume fraction and density weighted cortical thickness measured from HR‐QCT increased by 28.4% ± 7.6% and 9.6% ± 1.3% and trabecular spacing decreased by 16.0% ± 4.1%.

Genant and colleagues furthermore report that no change in integral vertebral volume was detected,^(^ ^9^ ^)^ which suggests that most of the cortical changes occurred at the endocortical envelope. However, not finding any significant changes with these modalities in the integral volume does not exclude periosteal bone apposition. Further evidence that romosozumab results in predominantly endocortical apposition was revealed in histological studies.^(^ ^4^, ^5^, ^6^ ^)^ In the histomorphometry study of Chavassieux and colleagues,^(^ ^5^ ^)^ we can see that the mineralizing surface (MS/BS) after 2 months of romosozumab increased to 24.59% of the endocortical surface, compared with 5.64% of the trabecular bone surface. Although the percentage increase of the MS/BS in the two regions is similar (393% at the endocortical surface compared with 374% at the trabecular bone), this does suggest that the anabolic action of romosozumab has the most effect at the endocortical surface, where a large percentage of the endocortical surface is already covered by bone formation.

By decomposing the thickness changes into a cortical and endocortical component, which describes the size of the transitional region from cortical bone to trabecular bone, we were able to provide an independent measure of the endocortical bone changes. Accordingly, we show a significant increase of 137.6% and 47.5% for romosozumab and teriparatide, respectively. Although this supports the presence of endocortical apposition, our approach can still not exclude the existence of periosteal apposition.

Furthermore, it should be noted that, even though we can see a large percentage increase in the Ec.Th, which is approximately 10 times that of the Ct.Th, the baseline values of the Ec.Th are approximately 10 times smaller. This means that, despite the greater percentage increase compared with the Ct.Th, the Ec.Th contributes approximately equally to the overall mass increase. At present, the relationship between the individual cortical parameters and the strength of the vertebrae remains to be determined. Recent advances in finite element analysis (FEA) might shed light on this by being able to simulate expected forces on the cortex, which could enhance FEA approaches. Here the proposed technique may aid in generating the geometric model by providing accurate measures of the vertebral cortex. This may subsequently inform to what extent the various therapies might influence the fracture risk.

A recent trial showed the clinical efficacy of romosozumab in women with severe osteoporosis by reducing vertebral fractures by 37% compared with alendronate within 12 months,^(^ ^33^ ^)^ as well as showing an increased strength in the lumbar spine as assessed by finite element analysis.^(^ ^34^ ^)^ In a separate trial of milder osteoporosis where 18% of participants had vertebral fractures, romosozumab was also found to be effective at preventing vertebral fracture^(^ ^35^ ^)^ and the magnitude and rapidity of the reduction in clinical vertebral fractures was notable.^(^ ^36^ ^)^ Understanding the localized variation in thickness is critical to studies of fracture initiation and prevention.^(^ ^37^, ^38^ ^)^ Considering the loading conditions leading to a fracture in the lumbar spine when biomechanically modeled, it is clear that certain anterior parts of the vertebra are particularly susceptible to failure. It follows that there is great interest in the location of bone accrual with treatment. In previous studies, we have found focal increases of the cortical thickness at load‐bearing regions of the proximal femur in response to teriparatide.^(^ ^28^ ^)^ Here, we see a region of increased thickness predominantly around the load‐bearing vertebral body in response to teriparatide. This could be due to the mechanism of action of teriparatide whereby the remodeling process present in loaded regions is enhanced. It is important to remember that our measurement point after 12 months of teriparatide treatment is only two‐thirds to halfway through the licensed course. Romosozumab, on the other hand, resulted in large and significant bone increases across the entire vertebra, including the vertebral shell and endplates, both of which are susceptible to fragility fractures. The posterior elements also showed improvements in cortical parameters.

A limitation of this study is the relatively small sample size in each treatment group. However, this study is of sufficient statistical power to observe significant global and local changes and differences between treatment groups. Although the increases in all of these parameters are a good indication of the vertebrae being reinforced with new bone, the role and contribution of the cortical and trabecular bone to the strength of vertebral bodies is still subject to debate. Further study is required to be able to relate these changes to the risk of fracture. Finally, it is important to note that the proposed method is not able to accurately determine the presence or absence of periosteal apposition in response to the various therapies. Neither is it able to distinguish a reduction in mineralization from an increase in porosity due to the resolution of clinical CT scans. These are important considerations when interpreting the results and comparing these results with other studies.

To conclude, this study provides new insights into the cortical and trabecular bone changes in response to romosozumab and teriparatide and treatment. Both were associated with large and significant increases in cortical and cancellous bone at the vertebrae. At 12 months, romosozumab led to statistically significantly larger gains compared with teriparatide. This study furthermore demonstrates the locations of bone accrual for these two anabolic agents, which are in regions important for vertebral strength, thereby providing further evidence to support their effectiveness in reducing the risk of fracture.

## Disclosures

KESP and GMT are inventors on a related patent application GB0917524.1, “Image data processing systems.” This does not alter the authors' adherence to policies on sharing data and materials. KESP has on behalf of Cambridge Enterprise (CU Enterprise) lectured in educational fora for Amgen and Lilly; has been an advisory board member for Amgen Inc. and UCB Pharma (all honoraria given by CU Enterprise to registered charities); and has previously received research grant funding from Amgen Inc. and Lilly. GMT has received research grant support from Amgen Inc. and Lilly. AHG has received research grant support from Amgen Inc. and Lilly. JPB has received research support from Mereo BioPharma, Radius Health, and Servier; has served as a consultant for Amgen and Servier; and has served on speakers' bureaus for Amgen. TW has lectured for UCB in educational fora and received research grant support from Amgen Inc. and Lilly.

### Peer review

The peer review history for this article is available at https://publons.com/publon/10.1002/jbmr.4465.

## Supporting information


**Appendix** **S1.** Supplemental Materials and Methods.

**Supplemental Fig. S1.** The average cortical thickness in 20 vertebrae, from clinical CT (*A*) and high‐resolution micro‐CT (*B*).

**Supplemental Fig. S2.** Validation plot of vertebral cortical thickness from paired high‐resolution micro‐CT and clinical resolution CT scans of 20 vertebrae. The cortical thickness was measured at 36,000 corresponding locations in the micro‐CT and clinical CT scan pairs. Each box in the grid shows the number of measurements where the thickness measured in the clinical CT scans corresponds to the value range in the *x* axis and the thickness measurement at the same location on the micro‐CT scans corresponds to the value range in *y* axis. The number of measurements is represented in the plot by the intensity of grayscale from many (black) to few (light gray) normalized in the diagonal direction. In the ideal case, all the black squares lie on the diagonal (white line) with all the measurements having the same values between the micro‐CT and clinical CT scans. The horizontal and vertical density plots show the number of measurements within each 0.05‐mm thickness range for high‐resolution micro‐CT (vertical) and clinical CT (horizontal). Here the grayscale values range between 0 for white and >5000 for black.

**Supplemental Fig. S3.** Absolute changes from baseline after 12‐month treatment of teriparatide measured by cortical bone mapping. Ct.BMD is not displayed because of the lack of regions with significant changes. Light gray regions had no statistically significant changes with time. Dark gray regions of the spinous processes and pedicles were not examined for endocortical and cancellous parameters.

**Supplemental Fig. S4.** Absolute changes from baseline after 12‐month treatment of romosozumab measured by cortical bone mapping. Ct.BMD is not displayed because of the lack of regions with significant changes. Light gray regions had no statistically significant changes with time. Dark gray regions of the spinous processes and pedicles were not examined for endocortical and cancellous parameters.

## Data Availability

Data available on request from the authors
