# Supplementary material for: Romosozumab Enhances Vertebral Bone Structure in Women With Low Bone Density
Source: J Bone Miner Res. 2021 Dec 16;37(2):256–64. doi: 10.1002/jbmr.4465 (PMC9299688; doi:10.1002/jbmr.4465)
Supplement: Supplementary file 1 — Appendix S1. Supplemental Materials and Methods. [file JBMR-37-256-s002.docx]

**Supplemental 1**

**Cortical parameter validation of the vertebrae**

While the cortical parameter measurements from clinical CT have been rigorously evaluated against micro-CT for the proximal femur, a new evaluation had to be performed for the vertebrae to ensure the reliability of the results. To validate whole vertebral body cortical thickness measurements in vivo, 20 dissections of whole vertebral bodies were imaged with clinical CT and high-resolution micro-CT. The medical history of each donor was available and confirmed as free from medical therapies that could have altered the bone tissue and from metabolic pathologies. Samples were from 6 older individuals: 3 females and 3 males aged between 53 and 79 with a mean (± standard deviation)age of 71±9.6 yrs, a mean height of 170±14.2 cm and mean weight of 79±30 kg. These samples include 15 lumbar vertebrae (L1-L5) and 6 lower thoracic (T10-T12) vertebrae. Technicians removed soft tissue and vertebral processes and the vertebral bodies were submerged in saline and vacuum packed to remove air bubbles prior to scanning. The vertebrae were scanned using the Brilliance 16 CT scanner (Phillips, Germany) at 120 kV and reconstructed with a slice thickness of 0.75 mm. Micro-CT scans were subsequently acquired of the same vertebrae wrapped in plastic and scanned in air using the SkyScan 1176 (Bruker, Belgium) scanner at 80kV, reconstructed with 0.35mm cubic voxels. All vertebrae were subsequently segmented in the clinical CT scans, and the meshes were registered to the corresponding vertebrae in the micro-CT data. In this way the same meshes (with identical vertex placements) were used to sample both datasets. Using Stradwin v5.4 (now available as Stradview, see https://mi.eng.cam.ac.uk/Main/StradView), measurements of the cortical thicknesses were taken as described in detail previously [17,18,28]. Supplemental Figure 1 shows the average of all 20 high resolution vertebral thickness measurements displayed on a single vertebral body. The global mean error (± standard deviation) for the CtTh measurements was 0.0430 ± 0.6163 mm. By matching the thickness measurements of 36,000 vertices, a validation plot of corresponding individual high-res and clinical resolution CtTh measurements was created (Supplemental Figure 2).

**Supplemental Figure 1.** The average cortical thickness in n=20 vertebrae, from clinical CT (A) and high resolution micro-CT (B).

**Supplemental Figure 2**. Validation plot of vertebral cortical thickness from paired high resolution micro-CT and clinical resolution CT scans of 20 vertebrae. The cortical thickness was measured at 36,000 corresponding locations in the micro-CT and clinical CT scan pairs. Each box in the grid shows the number of measurements where the thickness measured in the clinical CT scans corresponds to the value range in the x-axis and the thickness measurement at the same location on the micro-CT scans corresponds to the value range in y-axis. The number of measurements is represented in the plot by the intensity of greyscale from many (black) to few (light grey) normalised in the diagonal direction. In the ideal case all the black squares lie on the diagonal (white line) with all the measurements having the same values between the micro-CT and clinical CT scans. The horizontal and vertical density plots show the number of measurements within each 0.05mm thickness range for high resolution micro-CT (vertical) and clinical CT (horizontal). Here the greyscale values range between 0 for white and >5000 for black.

**Supplemental Figure 3.** Absolute changes from baseline after 12-month treatment of teriparatide measured by cortical bone mapping. CtBMD is not displayed because of the lack of regions with significant changes. Light grey regions had no statistically significant changes with time. Dark grey regions of the spinous processes and pedicles were not examined for endocortical and cancellous parameters.

**Supplemental Figure 4.** Absolute changes from baseline after 12-month treatment of romosozumab measured by cortical bone mapping. CtBMD is not displayed because of the lack of regions with significant changes. Light grey regions had no statistically significant changes with time. Dark grey regions of the spinous processes and pedicles were not examined for endocortical and cancellous parameters.
