# Supplementary figures and images for: Romosozumab Enhances Vertebral Bone Structure in Women With Low Bone Density
Source: J Bone Miner Res. 2021 Dec 16;37(2):256–64. doi: 10.1002/jbmr.4465 (PMC9299688; doi:10.1002/jbmr.4465)

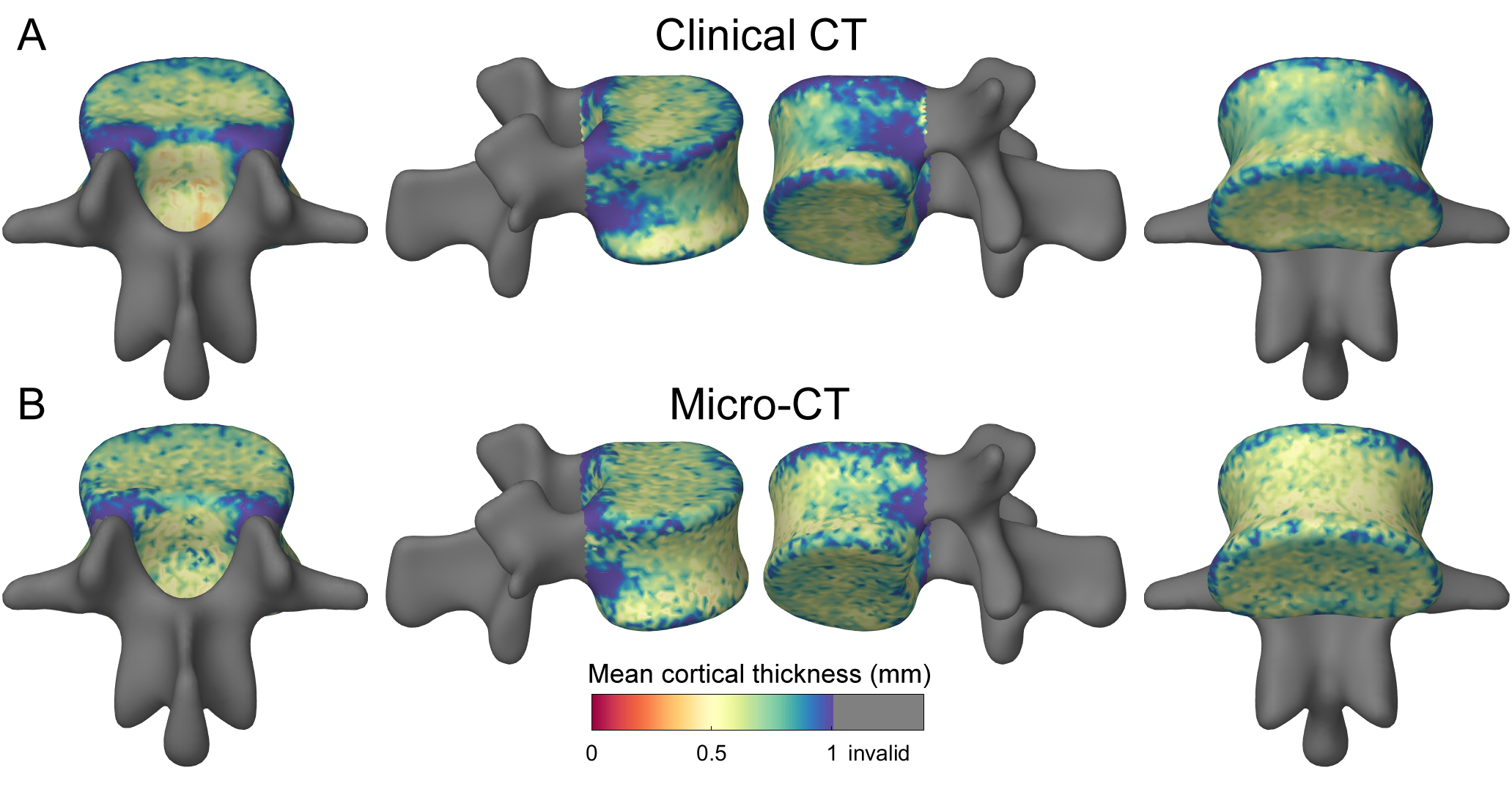

Supplement: Supplementary file 2 — Supplemental Fig. S1. The average cortical thickness in 20 vertebrae, from clinical CT (A) and high‐resolution micro‐CT (B). [file JBMR-37-256-s004.tif]

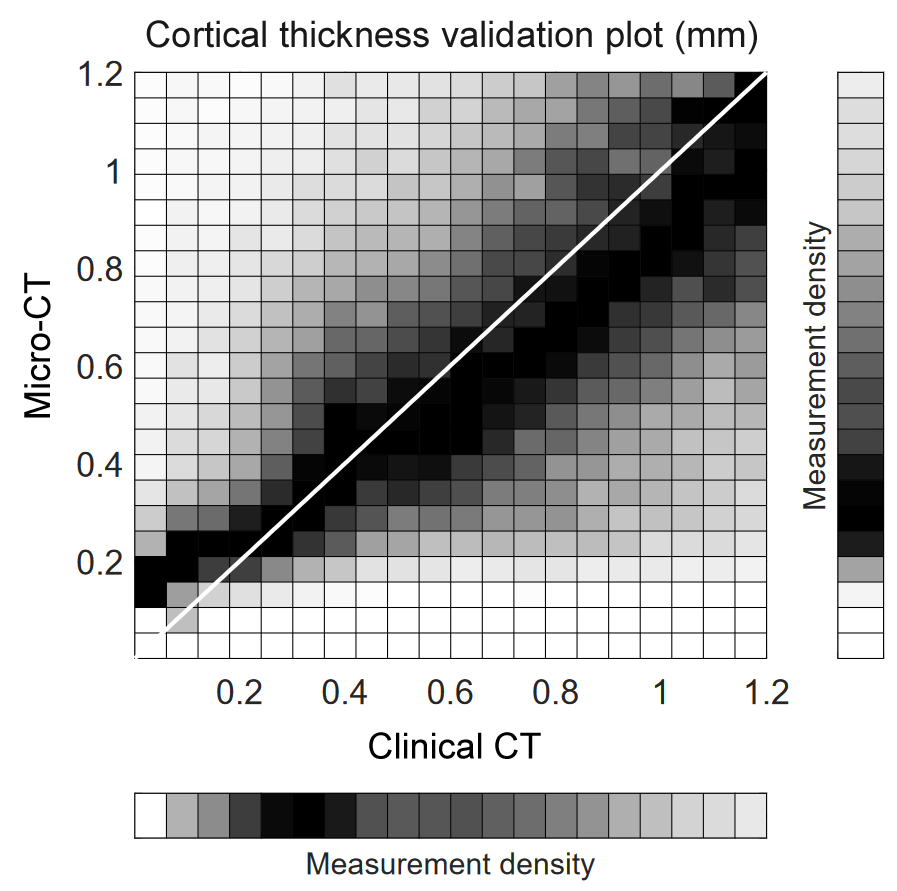

Supplement: Supplementary file 3 — Supplemental Fig. S2. Validation plot of vertebral cortical thickness from paired high‐resolution micro‐CT and clinical resolution CT scans of 20 vertebrae. The cortical thickness was measured at 36,000 corresponding locations in the micro‐CT and clinical CT scan pairs. Each box in the grid shows the number of measurements where the thickness measured in the clinical CT scans corresponds to the value range in the x axis and the thickness measurement at the same location on the micro‐CT scans corresponds to the value range in y axis. The number of measurements is represented in the plot by the intensity of grayscale from many (black) to few (light gray) normalized in the diagonal direction. In the ideal case, all the black squares lie on the diagonal (white line) with all the measurements having the same values between the micro‐CT and clinical CT scans. The horizontal and vertical density plots show the number of measurements within each 0.05‐mm thickness range for high‐resolution micro‐CT (vertical) and clinical CT (horizontal). Here the grayscale values range between 0 for white and >5000 for black. [file JBMR-37-256-s001.tif]

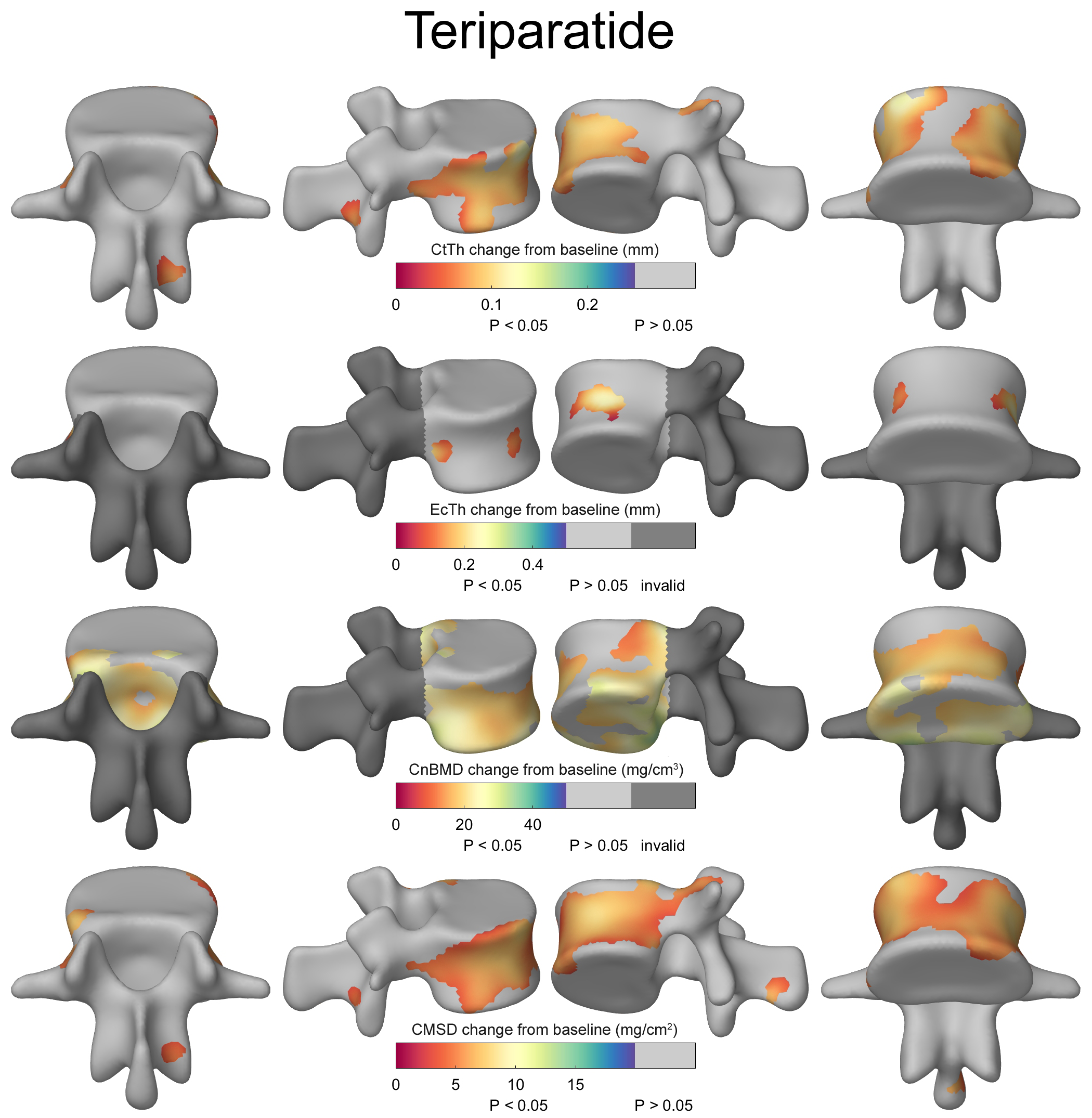

Supplement: Supplementary file 4 — Supplemental Fig. S3. Absolute changes from baseline after 12‐month treatment of teriparatide measured by cortical bone mapping. Ct.BMD is not displayed because of the lack of regions with significant changes. Light gray regions had no statistically significant changes with time. Dark gray regions of the spinous processes and pedicles were not examined for endocortical and cancellous parameters. [file JBMR-37-256-s005.tif]

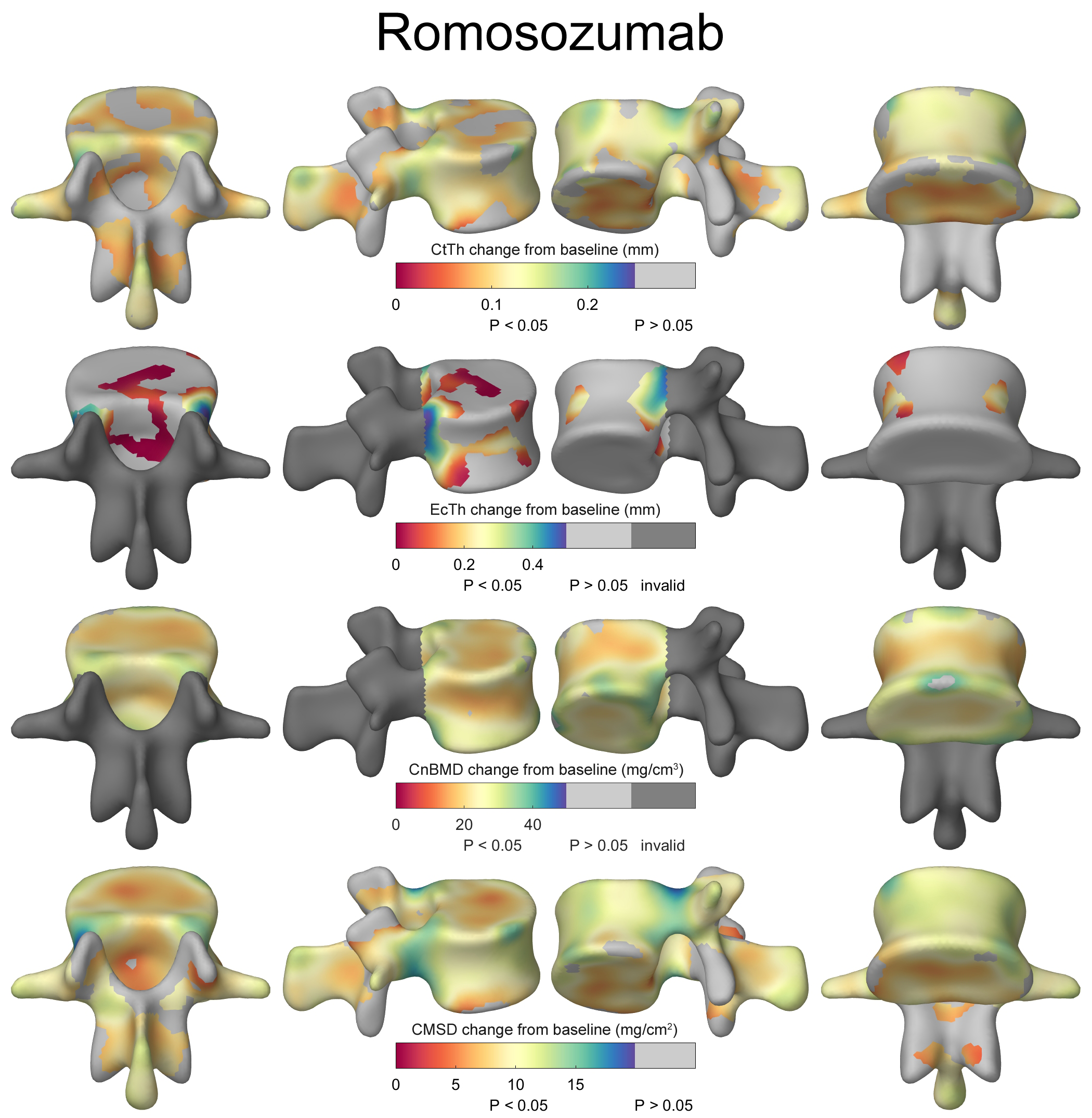

Supplement: Supplementary file 5 — Supplemental Fig. S4. Absolute changes from baseline after 12‐month treatment of romosozumab measured by cortical bone mapping. Ct.BMD is not displayed because of the lack of regions with significant changes. Light gray regions had no statistically significant changes with time. Dark gray regions of the spinous processes and pedicles were not examined for endocortical and cancellous parameters. [file JBMR-37-256-s003.tif]
